# Meat Adulteration in the MENA and GCC Regions: A Scoping Review of Risks, Detection Technologies, and Regulatory Challenges

**DOI:** 10.3390/foods14213743

**Published:** 2025-10-31

**Authors:** Zeina Daher, Mahmoud Mohamadin, Adem Rama, Amal Salem Saeed Albedwawi, Hind Mahmoud Mahaba, Sultan Ali Al Taher

**Affiliations:** 1 Faculty of Health Sciences, Higher Colleges of Technology, University City, Sharjah P.O. Box 7947, United Arab Emirates; arama@hct.ac.ae; 2 College of Veterinary Medicine, University of Al Dhaid, Sharjah P.O. Box 27272, United Arab Emirates; m.mohamadin@uodh.ac.ae; 3 Food Safety Department, Dubai Municipality, Dubai P.O. Box 67, United Arab Emirates; 4 Dubai Central Laboratory Department, Dubai Municipality, Dubai P.O. Box 67, United Arab Emirates

**Keywords:** meat adulteration, halal authenticity, PCR, biosensors, spectroscopy, MENA, GCC, food fraud, regulatory frameworks, public health

## Abstract

Background: Meat adulteration poses serious public health, economic, and religious concerns, particularly in the Middle East and North Africa (MENA) and Gulf Cooperation Council (GCC) regions where halal authenticity is essential. While isolated studies have reported undeclared species in meat products, a comprehensive regional synthesis of prevalence, detection technologies, and regulatory responses has been lacking. Methods: This scoping review followed PRISMA-ScR guidelines. A systematic search of PubMed, Scopus, and Web of Science from database inception to 15 September 2025 was conducted using controlled vocabulary (MeSH) and free-text terms. Eligible studies included laboratory-based investigations of meat adulteration in MENA and GCC countries. Data were charted on study characteristics, adulteration types, detection methods, and regulatory context. Results: Out of 50 records screened, 35 studies were included, covering 27 MENA/GCC countries. Prevalence of adulteration varied widely, from 5% in UAE surveillance studies to 66.7% in Egyptian native sausages. Undeclared species most frequently detected were poultry, donkey, equine, pig, and dog. Molecular methods, particularly PCR and qPCR, were most widely applied, followed by ELISA and spectroscopy. Recent studies introduced biosensors, AI-assisted spectroscopy, and blockchain traceability, but adoption in regulatory practice remains limited. Conclusions: Meat adulteration in the MENA and GCC regions is localized and product-specific rather than uniformly widespread. Detection technologies are advancing, yet regulatory enforcement and halal-sensitive verification remain fragmented. Strengthening laboratory capacity, harmonizing regional standards, and investing in portable biosensors, AI-enhanced spectral tools, and blockchain-based traceability are critical for consumer trust, halal integrity, and food safety.

## 1. Introduction

Meat adulteration, a critical form of food fraud, involves the intentional misrepresentation, substitution, or addition of undeclared components in meat products to increase profit margins. This practice threatens food authenticity, consumer trust, and public health globally. However, in Muslim-majority regions such as the Middle East and North Africa (MENA) and the Gulf Cooperation Council (GCC), the implications extend beyond economics and safety into deeply rooted religious and cultural spheres.

Globally, meat adulteration scandals, such as the European horse meat crisis of 2013, have exposed systemic weaknesses in food supply chains and highlighted the need for robust detection and regulation mechanisms [1,2]. In contrast, the MENA and GCC regions face unique challenges due to religious dietary restrictions, particularly the consumption of halal-certified meat. Here, adulteration not only introduces public health risks, such as allergen exposure and microbial contamination but also violates Islamic dietary laws when prohibited species like pork or dog meat are introduced into the food chain [3].

The rapid expansion of food markets in the MENA and GCC regions, combined with high import reliance, fragmented regulatory oversight, and underdeveloped traceability systems, has increased the likelihood of food fraud incidents [4]. Moreover, the complexity of halal certification, which must verify species, slaughter method, and processing integrity, adds layers of vulnerability if fraud is not detected effectively [5].

This review is warranted by the increasing reports of adulterated meat in regional markets and the emerging application of molecular, spectroscopic, and immunological technologies to detect such fraud. While global frameworks for meat authentication exist, the MENA and GCC regions require context-specific evaluations that consider religious, logistical, and regulatory factors. Thus, this review aims to (1) synthesize existing literature on the types and prevalence of meat adulteration in the MENA and GCC regions, (2) evaluate detection methodologies with respect to regional applicability, (3) assess regulatory frameworks and challenges, and (4) propose future directions to ensure halal integrity and food safety.

## 2. Materials and Methods

### 2.1. Protocol and Reporting

This scoping review adheres to PRISMA-ScR and includes a PRISMA 2020 flow diagram. While systematic reviews typically include a risk-of-bias assessment, scoping reviews focus on mapping and describing existing literature, thus critical appraisal is optional and was not conducted in this review [6,7].

### 2.2. Information Sources and Search Strategy

A comprehensive literature search was performed in PubMed, Scopus, and Web of Science from database inception to 15 September 2025. The search strategy combined controlled vocabulary (e.g., MeSH terms) and free-text terms related to “meat adulteration,” “species substitution,” “DNA detection,” and “MENA/GCC”. The literature search retrieved studies published between 2006 and 2025, providing nearly two decades of data on meat adulteration in the MENA and GCC regions. Detailed search strings for each database are provided in Supplementary File S1.

### 2.3. Eligibility Criteria

Eligibility Population, Concept, Context (PCC): Population/Phenomena: meat and meat products in MENA/GCC; Concept: adulteration/authentication (prevalence, methods, halal integrity, regulation); Context: MENA/GCC markets.

Studies were eligible if they

1. Reported on the detection of meat adulteration, mislabeling, or undeclared species in raw or processed meat products.
2. Included data from countries in the Middle East and North Africa (MENA) or Gulf Cooperation Council (GCC).
3. Used laboratory-based methods (e.g., PCR, ELISA, spectroscopy, or histology) for detection.

Exclusion criteria were:

- Studies outside the target region,
- Reviews, commentaries, or conference abstracts without primary data,
- General food safety studies not specifically addressing meat adulteration,
- Insufficient methodological detail to assess validity.

Only peer-reviewed articles in English were considered, which introduces a potential publication and language bias. In addition, gray literature and non-indexed regional reports were not included, which may have led to the omission of relevant local findings.

### 2.4. Study Selection

All identified records were imported into Mendeley Reference Manager for duplicate removal. Two reviewers independently screened titles and abstracts, followed by full-text review. Discrepancies were resolved through discussion.

**Figure 1:**
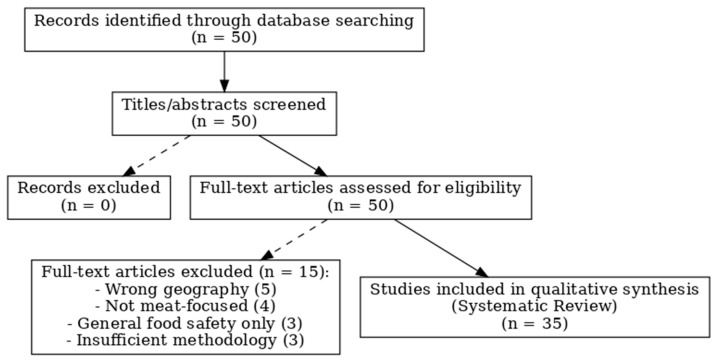
PRISMA-ScR flow diagram of study selection.

In total, 50 records were screened. After full-text review, 15 studies were excluded: wrong geography (*n* = 5), not meat-focused (*n* = 4), general food safety only (*n* = 3), and insufficient methodology (*n* = 3). The final review included 35 studies. The selection process is summarized in Figure 1 (PRISMA 2020 flow diagram).

### 2.5. Data Extraction

From each study, data were extracted on country, meat type, sample size, detection method, target species, prevalence of adulteration, and key findings. Extraction was performed by one reviewer and verified by a second. A summary table of included studies is provided in Table 1.

While scoping reviews do not typically perform formal bias appraisal, it is important to note that methodological robustness varied across the included studies. Most molecular assays (e.g., PCR/qPCR) were highly specific and reproducible, but others relied on histological or immunoassay-based methods with limited validation. Small sample sizes and inconsistent protocols further constrain generalizability. These methodological differences should be considered when interpreting prevalence patterns across the region.

## 3. Forms and Patterns of Meat Adulteration

Meat adulteration in the MENA and GCC regions manifests in several deceptive forms, including species substitution (e.g., replacing beef with poultry, equine, or donkey meat), incorporation of undeclared animal by-products such as offal or skin, and mislabeling regarding religious certification. Particularly concerning are cases where pork, dog, or donkey meat has been introduced into products labeled as halal or beef-based, violating both consumer rights and religious dietary laws.

Empirical studies have confirmed that adulteration is widespread. In Egypt, multiple investigations using PCR-based methods reported high prevalence, with equine DNA detected in 20% of sausage samples [8] and dog meat identified in up to 66.7% of native sausage products [9]. Poultry substitution has also been frequently observed, reaching 62.5% in beef products in some surveys [17]. In Iran, PCR studies revealed undeclared poultry, pig, donkey, and horse meat in approximately 7–8% of processed products [10], while a more recent histology–PCR approach found substitution rates as high as 66% [14]. In the UAE, a market surveillance study documented undeclared species in 5% of processed meat samples [4]. In Turkey, ELISA-based testing reported poultry contamination in beef products ranging from 6.2% to 39.2% [15]. In Morocco, a multiplex PCR analysis of ground and processed beef found poultry substitution in one-third to nearly half of all tested products [16]. Notably, certain adulterants such as donkey and dog meat were detected at significant levels in Egypt, underscoring the seriousness of fraudulent practices. To enhance clarity, the results are presented in alignment with the four stated objectives of this review: prevalence and forms of adulteration, detection methodologies, regulatory frameworks, and future directions.

Several economic and structural drivers contribute to these practices. The significant price differential between premium meats (e.g., beef or lamb) and lower-cost substitutes (e.g., poultry, donkey, equine) incentivizes fraudulent substitution. The region’s dependence on imports, fragmented supply chains, and limited routine surveillance exacerbate systemic vulnerabilities in the meat sector [2,3].

Consumer awareness across the region also remains limited. While demand for halal integrity is strong, public access to transparent labeling and validated testing outcomes is often inadequate. Surveys in GCC countries suggest that many consumers remain unaware of the risk of adulteration, placing high trust in branding and halal certification despite occasional inconsistencies in regulatory oversight [5].

When viewed longitudinally, trends indicate an increased adoption of PCR-based methods after 2010, coinciding with a gradual expansion of documented adulteration cases. Prevalence appears consistently highest in Egypt across multiple time points, whereas UAE studies report lower levels (≈5%), reflecting relatively stronger regulatory controls.

**Figure 2:**
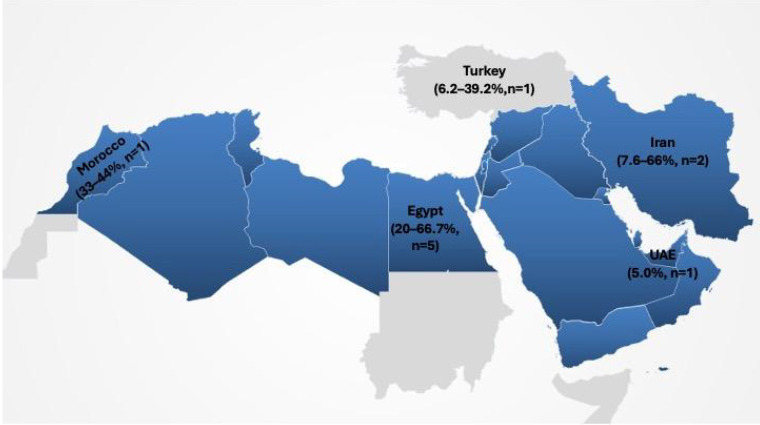
Reported prevalence of meat adulteration in MENA and GCC studies (2006–2025). Values indicate prevalence ranges and number of studies per country.

A graphical overview of reported prevalence by country is presented in Figure 2. The distribution of studies is uneven, with Egypt (*n* = 5) contributing the largest share of data, followed by Iran (*n* = 2), and single studies from the UAE (*n* = 1), Turkey (*n* = 1), and Morocco (*n* = 1) [4,8,9,10,11,15,16,17]. By contrast, no indexed prevalence surveys were identified for several North African countries (e.g., Algeria, Tunisia, Libya) or Levant states (e.g., Lebanon, Jordan, Syria, Yemen), nor for most GCC countries (Kuwait, Qatar, Oman, Bahrain, Saudi Arabia) within the review period. This imbalance underscores that large parts of the MENA/GCC remain underrepresented, limiting the generalizability of available findings and highlighting the need for broader surveillance across the region.

## 4. Detection Technologies

### 4.1. Conventional Techniques

Traditional approaches such as histology and physicochemical analyses were among the earliest tools for meat authentication. Histological examination can identify structural tissue differences and detect unauthorized animal parts, often combined with staining techniques [1]. Physicochemical assays, including protein profiling (SDS-PAGE), electrophoresis, and chromatography (HPLC, GC-MS), detect inconsistencies in composition (fat, protein, moisture) that may indicate adulteration [18]. While useful for screening, these methods are labor-intensive, require expertise, and cannot reliably differentiate species in complex or processed products.

### 4.2. Molecular Biology Methods

Molecular assays are considered the benchmark for meat authentication due to their sensitivity, specificity, and ability to detect trace adulterants. Polymerase Chain Reaction (PCR) has been widely used across MENA and GCC studies. Elshazly et al. [8] and Doosti et al. [10] applied PCR successfully to identify equine, canine, and poultry DNA in commercial samples. Variants such as quantitative PCR (qPCR) and TaqMan-based multiplex PCR permit simultaneous detection and quantification of multiple species, making them suitable for regulatory monitoring [3]. Other adaptations include PCR-RFLP for species differentiation [10], DNA barcoding using mitochondrial genes such as 12S rRNA [13], and loop-mediated isothermal amplification (LAMP) for rapid on-site testing [19]. Next-generation sequencing (NGS) has emerged as a powerful untargeted approach, enabling analysis of complex mixtures, though cost and data complexity currently restrict its application in routine practice [20].

### 4.3. Spectroscopic and Imaging Techniques

Spectroscopic tools offer rapid and non-destructive alternatives. Fourier Transform Infrared (FTIR) and Near-Infrared (NIR) spectroscopy generate spectral fingerprints that, when coupled with chemometric analysis, can differentiate species [21]. Raman spectroscopy provides high-resolution insights into protein and fat signatures, while hyperspectral imaging (HSI) combines spatial and spectral data to detect adulterants at pixel level [22]. Despite their advantages, these methods require extensive calibration and expensive instrumentation, which has limited their adoption in MENA/GCC settings where resources and region-specific calibration models remain limited.

### 4.4. Emerging and Integrated Technologies

Recent years have seen the rise of biosensors, digital traceability, and artificial intelligence. DNA and protein-based biosensors provide portable, user-friendly platforms capable of on-site detection, with potential applications in halal verification [23,24]. Blockchain technologies, often integrated with IoT devices, have been piloted by retailers such as Carrefour to secure supply chain traceability, offering tamper-proof documentation of origin and halal status [5,25]. Pilot applications have already been reported. Portable electrochemical and optical biosensors were tested in controlled studies to rapidly detect undeclared species DNA in processed meats, demonstrating high sensitivity and short turnaround times in market-like settings [24]. On the traceability side, blockchain frameworks have moved beyond conceptual design. For example, pilot halal supply chain models integrating blockchain with IoT sensors have been trialed to ensure cold-chain integrity and certification compliance [26]. More recently, dual blockchain architectures were evaluated for fresh-meat traceability, achieving secure transaction validation and proof-of-authority consensus in simulated halal market scenarios [27]. These cases highlight the transition from theory to practical demonstrations of biosensor- and blockchain-based meat authentication in MENA and GCC-relevant contexts. Artificial intelligence (AI) and machine learning (ML) are increasingly applied to spectroscopic datasets, improving classification accuracy and enabling automated carcass grading and anomaly detection [28,29,30]. Omics approaches, including proteomics and metabolomics, provide comprehensive molecular profiles of meat products [31] though they remain confined to research contexts due to cost and complexity.

A comparative summary of the main detection approaches is provided in Table 2**,** outlining their principles, advantages, limitations, and current applicability in MENA and GCC contexts.

### 4.5. Challenges in Implementation

Despite technological progress, several barriers limit the adoption of advanced detection tools in the MENA and GCC regions. First, the high cost of molecular and spectroscopic instruments, combined with the expense of reagents and maintenance, creates budgetary constraints, especially for smaller regulatory labs and local producers [4]. Second, a shortage of trained personnel with expertise in molecular diagnostics and chemometrics hampers method implementation and validation.

Additionally, many detection tools are developed in non-Muslim-majority countries and may not be aligned with halal-specific verification needs. For example, detecting species identity is necessary but not sufficient; verification must also address slaughter method, cross-contamination, and handling procedures. Another critical challenge is the lack of unified regulatory standards across the region, which hinders cross-border cooperation and mutual recognition of test results.

To overcome these obstacles, investment is needed in developing portable, cost-effective, and halal-sensitive diagnostic tools. Regional collaboration among regulatory agencies, academic institutions, and certification bodies can foster standardization, resource-sharing, and innovation in meat authentication technologies.

## 5. Public Health, Religious, and Economic Implications

### 5.1. Public Health Risks

The adulteration of meat products introduces a spectrum of public health risks. Microbial contamination is a primary concern, particularly when fraudulent meat includes offal or comes from diseased animals. Pathogens such as *Salmonella*, *E. coli*, and *Listeria monocytogenes* are frequently associated with improperly handled or unregulated meat, posing risks of severe gastroenteritis, hemolytic uremic syndrome, and even death. Inadequately processed meats may also harbor antibiotic-resistant bacteria, exacerbating the global antimicrobial resistance crisis [1].

Another pressing issue is the presence of allergens and chemical residues. Undeclared additives such as soy protein, dairy, or gluten-containing fillers can cause allergic reactions ranging from mild irritation to life-threatening anaphylaxis in sensitized individuals. Chemical adulterants, including preservatives, colorants, and residues of veterinary drugs, further compromise consumer safety and may exceed established maximum residue limits (MRLs), contributing to chronic toxicity over time [2].

### 5.2. Religious and Ethical Concerns

In the MENA and GCC regions, where halal observance is integral to daily life, meat adulteration takes on significant religious and ethical dimensions. The substitution of halal meat with non-halal alternatives such as pork, dog, or improperly slaughtered animals is not merely fraudulent but spiritually offensive. Numerous studies, including Hamouda et al. [11] and Elshazly et al. [8], have revealed adulteration rates of up to 66.7% in so-called halal-labeled products. These violations undermine public trust in certification systems, challenge the credibility of Islamic regulatory bodies, and trigger social backlash.

Consumers often rely heavily on halal logos and certification seals, yet these symbols may not always reflect true compliance due to inconsistent enforcement and fragmented halal governance. Public outrage in several documented cases has led to product recalls, reputational damage, and policy debates on certification reform [5]. Strengthening halal auditing, standardizing procedures across regions, and using sensitive analytical methods like PCR and blockchain-backed certification are essential to safeguarding consumer faith and religious dignity.

### 5.3. Economic and Legal Implications

Beyond its health and religious consequences, meat adulteration also generates significant economic losses and legal ramifications. Food fraud disrupts fair market competition, penalizes compliant producers, and undermines consumer confidence. Deliberate mislabeling of meat products as halal or premium beef allows unscrupulous actors to profit unfairly at the expense of legitimate businesses.

At a macroeconomic level, fraudulent practices can lead to import bans, international trade restrictions, and damaged reputations of exporting countries. For instance, the detection of undeclared species in exported products has led to border rejections and intensified scrutiny from regulatory authorities abroad. Legally, penalties for mislabeling and fraud vary across the region and are often insufficient to deter future violations. The lack of harmonized legal frameworks within the GCC enables regulatory gaps that are exploited across borders [4].

To address these issues, countries must invest in strengthening food laws, improving surveillance, and adopting internationally recognized standards. Legal harmonization, especially regarding definitions of food fraud and halal compliance, is vital for regional trade integrity and consumer protection.

## 6. Regulatory Frameworks and Enforcement in MENA and GCC

### 6.1. Current Regulations in MENA and GCC

Meat adulteration regulation in the MENA and GCC regions is governed by a mosaic of country-specific laws, halal certification bodies, and food safety authorities. In the UAE, food safety and halal certification are managed by the Emirates Authority for Standardization and Metrology (ESMA) in conjunction with the Ministry of Climate Change and Environment (MoCCAE). The UAE mandates halal certification for all imported meat, with periodic updates to national standards such as UAE.S GSO 993:2015 [32]. Saudi Arabia operates through the Saudi Food and Drug Authority (SFDA), which ensures that all imported meat meets halal slaughter requirements and is traceable via the ZAD electronic import system.

In Egypt, the National Food Safety Authority (NFSA) oversees meat quality and halal integrity, although halal certification is often outsourced to Islamic centers abroad. Jordan, Kuwait, and Qatar each have their own food safety departments, but their enforcement capacity varies depending on infrastructure and political will. Regional efforts are supported by the Gulf Standardization Organization (GSO), which issues harmonized standards such as GSO 2055-1: Halal Food—General Requirements [33]. However, actual implementation and enforcement of these standards differ significantly between member states [5].

### 6.2. Comparison with International Standards

Compared to international frameworks such as the European Union’s General Food Law (Regulation EC No 178/2002) [34], Codex Alimentarius guidelines, and USDA FSIS standards, the MENA and GCC systems often lack standardized testing requirements and consistent labeling laws. The EU requires accurate species labeling and mandates DNA-based testing for suspected fraud, supported by the Rapid Alert System for Food and Feed (RASFF). Similarly, the USDA employs robust verification and traceback protocols, including NIMS and laboratory auditing of meat authenticity [2].

ISO standards, such as ISO 22000 [35] for food safety management and ISO 17025 [36] for laboratory accreditation, are increasingly adopted in GCC countries, yet enforcement remains sporadic. While the GSO references Codex and ISO guidelines, their legal application is often non-binding, and enforcement mechanisms lack the institutional robustness observed in Western regulatory bodies [23].

### 6.3. Challenges in Enforcement

Key challenges facing meat fraud enforcement in MENA and GCC regions include cross-border trade inconsistencies, porous borders, and the presence of informal meat markets that operate outside regulatory oversight. Many countries lack centralized databases or digital traceability systems, complicating recall procedures and undermining regulatory response capacity [4].

Additionally, laboratory limitations within national food control agencies remain a persistent obstacle. Many labs are underfunded, lack molecular detection capabilities, or suffer from shortages in trained personnel. In some countries, halal verification relies heavily on document inspection without supporting analytical testing. This gap exposes consumers to mislabeled or contaminated products and weakens trust in halal integrity [13].

Political instability and bureaucratic inefficiencies further compound these issues, particularly in conflict-affected countries like Yemen and Libya, where food fraud thrives in unregulated supply chains. Fragmentation between religious authorities and scientific regulators adds to enforcement complexity, making inter-agency cooperation difficult and inconsistent across borders [3].

In summary, while regional and national institutions have made strides in drafting halal and food fraud standards, the lack of harmonized enforcement, laboratory infrastructure, and transnational coordination continues to impede effective governance. Bridging this gap requires investment in modern detection technologies, regulatory capacity-building, and harmonization of halal standards with global food safety systems.

## 7. Comparative Observations

The MENA and GCC regions face a distinct set of challenges when it comes to preventing and detecting meat adulteration, particularly when compared with regulatory systems in Europe, North America, and Asia. In the European Union, the General Food Law (Regulation EC No 178/2002) mandates rigorous traceability, routine species verification, and consumer transparency. Tools such as the Rapid Alert System for Food and Feed (RASFF) enable immediate responses to non-compliance, and DNA-based species identification is routinely implemented in national reference laboratories [2].

In contrast, MENA and GCC nations often struggle with inconsistent implementation of regulations, limited laboratory infrastructure, and fragmented halal certification frameworks. Countries like the UAE and Saudi Arabia have made strides toward harmonizing halal and food safety regulations through agencies such as ESMA and SFDA, yet smaller or less resourced countries lack comprehensive systems to detect and prosecute food fraud [4].

While Malaysia has developed one of the world’s most recognized halal certification systems, incorporating religious, scientific, and legal validation, the MENA and GCC regions remain fragmented in their approach. Certification bodies differ in their testing requirements and religious interpretations, limiting international trust and creating difficulties for cross-border trade.

Globally, there is also a growing integration of blockchain and artificial intelligence in food traceability systems. For instance, the IBM Food Trust and initiatives in the Netherlands have piloted blockchain to track meat origin from farm to fork. These technologies have yet to be widely adopted in the MENA and GCC regions, where digital transformation of supply chains remains in early stages [5].

Despite these limitations, there are positive developments. Regional institutions are increasingly aware of the economic and religious costs of meat fraud, and there is a shift toward adopting ISO-aligned laboratory standards, enhancing inspector training, and participating in global food safety dialogues. Collaborative projects and information-sharing across borders will be essential to reach international equivalency.

## 8. Knowledge Gaps and Research Needs

Despite growing attention to meat adulteration in MENA and GCC regions, several critical knowledge gaps persist. First, there is a lack of comprehensive, up-to-date surveillance data on the prevalence of meat fraud across different countries and food categories. Most published studies are case-specific, focused on small sample sizes, and lack longitudinal data to assess trends over time [13].

Second, the scientific validation of halal status remains underexplored. While DNA-based methods are excellent for species identification, there is a need for research into technologies that can verify slaughter methods and prevent cross-contamination during processing. Biosensors and LAMP-based assays hold promise but require further adaptation and validation for halal-specific use cases [19,31].

Third, there is insufficient exploration of consumer attitudes toward halal integrity and adulteration risks. Behavioral research and surveys across different socio-demographic groups in the region would shed light on trust in certification, risk perception, and willingness to pay for verified products, important data for shaping policy and awareness campaigns [5].

Fourth, more interdisciplinary work is needed that brings together molecular biologists, religious scholars, regulatory officials, and social scientists. Such collaboration would aid in the development of integrated frameworks for authentication, certification, and enforcement tailored to the region’s religious and cultural context.

Finally, research funding, particularly from regional sources, is currently limited. Creating grant programs that prioritize food fraud detection, halal science, and regulatory innovation can catalyze academic engagement and capacity building.

Addressing these gaps is essential for designing robust, science-driven, and culturally sensitive food fraud mitigation strategies in the MENA and GCC regions.

## 9. Future Directions and Recommendations

The complexity of meat adulteration in the MENA and GCC regions demands a multi-pronged strategy that couples cutting-edge analytics with regulatory harmonization and halal-specific verification. The priorities below reflect where the evidence base points most strongly.

Advance laboratory and analytical capacity beyond PCR/ELISA.

National and regional labs should augment routine PCR with high-resolution/untargeted platforms (e.g., metabolomic/volatilomic readouts, HRMS) and broader *foodomics* workflows to capture complex adulteration signatures that targeted assays may miss. Recent reviews show that integrated *omics* approaches materially improve food authentication, traceability, and screening performance in meat systems [37,38].

Adopt halal-sensitive, portable biosensors for field use.

To complement centralized testing, validated biosensing solutions (DNA/protein immunosensors; electrochemical genosensors) can deliver rapid, on-site species checks, including pork detection in beef-labeled products, thereby aligning with halal verification needs during inspections and market surveillance [24].

Leverage AI with advanced spectroscopy for rapid screening.

Evidence now demonstrates that AI-assisted Raman/hyperspectral pipelines can accurately classify meat species and detect mixing/adulteration, offering fast triage before confirmatory molecular testing. Methodological work also maps the path from targeted to non-targeted spectral screening (chemometrics and ML), which is essential for scalable fraud detection programs [28,30,39].

Implement blockchain-enabled digital traceability integrated with IoT.

For halal supply chains, blockchain architectures, when embedded within food safety management and device-level sensing, can strengthen provenance, certification integrity, and recall responsiveness. Recent syntheses across food sectors (with halal-focused frameworks emerging) underline feasibility, design choices, and adoption barriers; these should guide pilot deployments in GCC contexts [26,27,40].

Foster GCC-level regulatory harmonization and shared infrastructure.

Harmonized test requirements (method performance specs, confirmatory thresholds), shared reference materials, and cross-border recall protocols would reduce enforcement gaps. Coupling common analytical guidance (including non-targeted screens followed by confirmatory PCR/qPCR) with inter-laboratory exercises will accelerate equivalence and trust across the region [38]. Another measurable priority is the establishment of a GCC-level reference laboratory network dedicated to meat authentication, which could coordinate inter-laboratory proficiency testing and develop a unified halal authenticity database accessible to regulators and certification bodies across the region.

Strengthen legal deterrents and consumer engagement.

Fraud penalties should reflect economic and religious harm; public-facing transparency (e.g., QR-linked certificates, inspection dashboards) can raise trust and create market pressure for compliance, especially when paired with rapid field tests and traceability data [40].

Together, these actions align scientific innovation (omics, biosensors, AI-spectroscopy) with governance (blockchain traceability, harmonized standards), positioning MENA/GCC authorities to deliver faster detection, halal-sensitive verification, and credible cross-border enforcement.

## 10. Conclusions

Meat adulteration represents a multifaceted threat in the MENA and GCC regions, encompassing serious risks to public health, religious integrity, and economic sustainability. Although detection technologies such as PCR, qPCR, and spectroscopic tools are increasingly available, widespread implementation is hindered by regulatory fragmentation, limited infrastructure, and inconsistent enforcement.

This review highlighted the widespread nature of meat adulteration in the region, with documented cases of undeclared species, including pork, dog, and equine meat, in products marketed as halal. Detection techniques are advancing, but effective governance requires harmonized standards, reliable certification systems, and public accountability.

By integrating modern diagnostic tools, fostering regional regulatory cooperation, and embracing digital traceability technologies, MENA and GCC countries can take substantial steps toward protecting their food supply. Strengthening halal verification systems and aligning with international standards will not only safeguard consumer health and religious rights but also bolster trade competitiveness and public trust in the integrity of meat products.

Continued interdisciplinary research, policy innovation, and stakeholder engagement are essential for achieving a transparent, halal-compliant, and fraud-resilient meat sector in the MENA and GCC regions.

## Figures and Tables

**Table 1:** Summary of studies on meat adulteration in MENA/GCC (2006–2025).

Country	Sample Type (*n*)	Method	Findings	Reference
Egypt	Sausages, kofta, luncheon (*n* ≈ 100 across studies)	PCR, PCR-RFLP	Adulteration 20–66.7%; detection of donkey, dog, horse, poultry	[8,9,10,11,12,13]
Iran	Sausages, hamburgers, minced beef (*n* ≈ 80)	PCR, Histology–PCR	Adulteration 5–66%; detection of chicken, pork, donkey	[10,14]
UAE	Beef and lamb products (*n* ≈ 20)	PCR	Adulteration 5%; detection of pork traces in retail meat	[4]
Turkey	Meat products (sausages, salami, *n* ≈ 120)	ELISA	Adulteration 6.2–39.2%; detection of poultry proteins in beef-based products	[15]
Morocco	Ground beef (24), processed beef (16)	Multiplex PCR	33–44% adulteration with poultry (chicken and/or turkey)	[16]

**Table 2:** Summary of Detection Technologies for Meat Authentication in MENA/GCC.

Technology	Principle	Advantages	Limitations	Regional Applicability
PCR/qPCR	Amplification of species-specific DNA	High specificity, detects trace adulterants, widely validated	Requires laboratory infrastructure and trained staff	Routine use in Egypt, Iran, UAE, Morocco
ELISA	Antigen–antibody binding	Relatively low cost, simple to perform	Cross-reactivity, lower specificity than PCR	Used in earlier regional studies
Spectroscopy (FTIR, NIR, Raman)	Spectral fingerprinting of chemical bonds	Rapid, non-destructive, suitable for screening	Needs extensive calibration, costly equipment	Limited application in GCC due to resource constraints
Biosensors	Portable DNA/protein-based sensors	On-site testing, rapid results, halal-sensitive applications	Limited validation, early-stage adoption	Pilot projects emerging
Blockchain traceability	Tamper-proof digital ledger of supply chain	Enhances transparency, traceability, consumer trust	High implementation costs, interoperability challenges	Early pilots with retailers in GCC
Omics approaches	Proteomic, metabolomic, genomic profiling	High-resolution, comprehensive detection	Expensive, complex data analysis, research-focused	Currently research only, potential for future integration

## Data Availability

No new data were created or analyzed in this study. Data sharing is not applicable to this article.

## Supplementary Materials

The following supporting information can be downloaded at: https://www.mdpi.com/article/10.3390/foods14213743/s1. The full search strings for each database and the completed PRISMA-ScR checklist are provided in Supplementary File S1.

## Abbreviations

The following abbreviations are used in this manuscript:

AI	Artificial Intelligence
COD	Codex Alimentarius
DNA	Deoxyribonucleic Acid
ELISA	Enzyme-Linked Immunosorbent Assay
ESMA	Emirates Authority for Standardization and Metrology
EU	European Union
FTIR	Fourier Transform Infrared Spectroscopy
GC-MS	Gas Chromatography–Mass Spectrometry
GCC	Gulf Cooperation Council
GFSI	Global Food Safety Initiative
GSO	Gulf Standardization Organization
HSI	Hyperspectral Imaging
HPLC	High-Performance Liquid Chromatography
ISO	International Organization for Standardization
KSA	Kingdom of Saudi Arabia
LAMP	Loop-Mediated Isothermal Amplification
ML	Machine Learning
MoCCAE	Ministry of Climate Change and Environment (UAE)
MENA	Middle East and North Africa
MRL	Maximum Residue Limit
NGS	Next-Generation Sequencing
NIR	Near-Infrared Spectroscopy
PCA	Principal Component Analysis
PCC	Population, Concept, Context
PCR	Polymerase Chain Reaction
PCR-RFLP	Polymerase Chain Reaction–Restriction Fragment Length Polymorphism
PLS-DA	Partial Least Squares-Discriminant Analysis
qPCR	Quantitative Polymerase Chain Reaction
RASFF	Rapid Alert System for Food and Feed
RFID	Radio Frequency Identification
SFDA	Saudi Food and Drug Authority
UAE	United Arab Emirates
USDA	United States Department of Agriculture
